# COVID-19 Vaccination Coverage, Intent, Knowledge, Attitudes, and Beliefs among Essential Workers, United States

**DOI:** 10.3201/eid2711.211557

**Published:** 2021-11

**Authors:** Kimberly H. Nguyen, David Yankey, Kelsey C. Coy, Kathryn A. Brookmeyer, Neetu Abad, Rebecca Guerin, Girija Syamlal, Peng-jun Lu, Brittney N. Baack, Hilda Razzaghi, Andrea Okun, James A. Singleton

**Affiliations:** Centers for Disease Control and Prevention, Atlanta, Georgia, USA (K.H. Nguyen, D. Yankey, K.C. Coy, K.A. Brookmeyer, N. Abad, R. Guerin, G. Syamlal, P. Lu, B. Baack, H. Razzaghi, A. Okun, J.A. Singleton);; Leidos, Inc., Atlanta (K.C. Coy)

**Keywords:** respiratory infections, severe acute respiratory syndrome coronavirus 2, SARS-CoV-2, SARS, COVID-19, coronavirus disease, zoonoses, viruses, coronavirus, vaccination, intent, essential workers, perceptions, attitudes, United States

## Abstract

We assessed coronavirus disease vaccination and intent and knowledge, attitudes, and beliefs among essential workers during March–June 2021. Coverage was 67%; 18% reported no intent to get vaccinated. Primary concerns were potential side effects, safety, and lack of trust in vaccines, highlighting the importance of increasing vaccine confidence in this population.

Essential workers, who conduct a range of operations and services to ensure the continuity and viability of critical infrastructure functions, have more coronavirus disease (COVID-19) exposures and experience greater risk for severe illness and death than do nonessential workers (*1**–**4*). In December 2020, the US Advisory Committee on Immunization Practices issued recommendations prioritizing healthcare personnel (HCP), nonhealthcare frontline essential workers, and other essential workers for COVID-19 vaccination (*5*) (Appendix). Previous findings indicate that <50% of essential workers intended to get vaccinated: 37.1% in September 2020 and 49.1% in December 2020 (*6*,*7*). Assessing vaccination coverage and intent among essential workers, who continue to face increased risk because of their public-facing roles can help tailor messages and strategies to increase vaccination uptake and confidence among this high-risk group. We analyzed data from surveys to assess COVID-19 vaccine coverage and intent and knowledge, attitudes, and beliefs (KABs) among essential workers.

## The Study

We analyzed data from 2 nationally representative household surveys collected over 6 COVID-19 waves during March 5–June 2, 2021, Ipsos KnowledgePanel (*8*) and NORC AmeriSpeak (*9*) (Appendix). Because of the small sample sizes, to bolster the strength of the study’s estimates and increase the reliability of results, we combined data for analysis from each survey during the 6 waves of data collection.

The total sample size was 7,734 respondents; 5,303 were essential workers and 2,426 nonessential workers. We used the American Association for Public Opinion Research definition for cooperation rates (*10*), the proportion of all respondents interviewed of all eligible units ever contacted. Among respondents, cooperation rates were 20.3%–60.1%.

We categorized respondents as essential or nonessential workers. The essential worker category comprised the HCP, nonhealthcare frontline, and other essential worker groups (Appendix). We examined sociodemographic characteristics, including age group, sex, race and ethnicity, annual household income, health insurance status, marital status, urban versus rural status, and underlying conditions (Appendix).

We assessed vaccination status, intent, and KABs by worker group (Appendix). We categorized respondents as reachable or reluctant; reachable respondents said they probably would get or were unsure about getting a vaccine, whereas reluctant respondents said they probably or definitely would not get a vaccine. We assessed the following KABs about COVID-19 vaccination: reasons for not getting vaccinated, barriers to getting vaccinated, motivators for getting vaccinated, concerns about getting vaccinated, and concerns about vaccine side effects.

We weighted all surveys to ensure US population representation (Appendix). We used contrast tests for differences in percentages to compare reachable versus reluctant groups among each of the worker categories. This activity was reviewed by the Centers for Disease Control and Prevention and was conducted consistent with applicable federal law and Centers for Disease Control and Prevention policy (Appendix).

**Figure 1:**
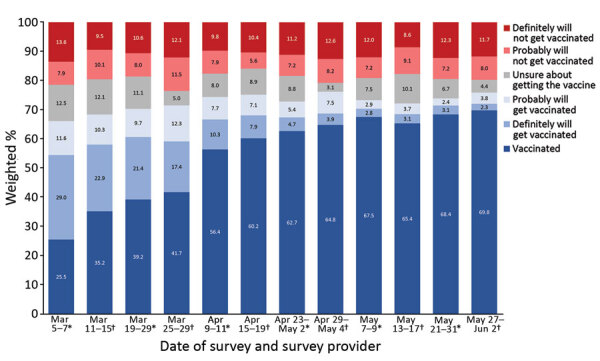
Trends in COVID-19 vaccination status and intent among essential workers, United States, March 5–June 2, 2021. *Data collected by Ipsos KnowledgePanel (*8*). †Data collected by NORC AmeriSpeak (*9*). COVID-19, coronavirus disease.

**Figure 2:**
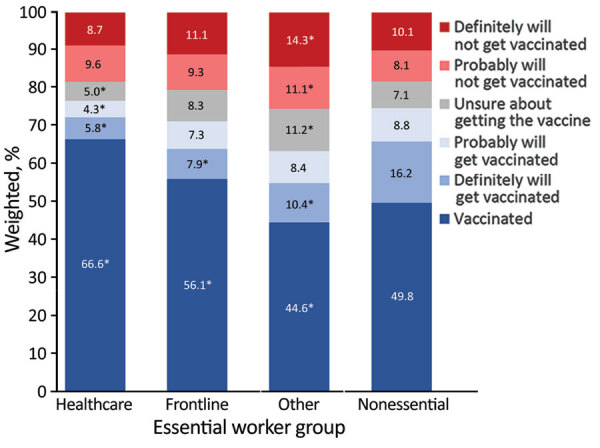
Average prevalence of COVID-19 vaccination status and intent by essential and nonessential worker groups, United States, March 5–June 2, 2021. Asterisk (*) indicates statistically significant (p<0.05) differences between vaccination coverage and intent among each essential worker group versus vaccination coverage and vaccination intent among nonessential workers. COVID-19, coronavirus disease.

Vaccination coverage among essential workers increased from 25.5% in March 2021 to 69.8% in June 2021 (Figure 1). Average vaccination coverage during the study period was higher for HCP (66.6%) and frontline essential workers (56.1%) and lower for other essential workers (44.6%) and nonessential workers (49.8%) (Figures 1, 2). The percentage of reluctant persons was lowest among HCP (18.3%) and highest among other essential workers (25.5%) (Figure 2). In addition, the percentage of reluctant adults was highest (25.0%) among persons 18–34 years of age; those who had a high school education or less (28.1%), income <$25,000 (26.3%), or no health insurance (32.3%); and those who lived in rural areas (29.3%) (Appendix Table 1).

Among all unvaccinated essential workers, reasons for not getting vaccinated included concern for possible side effects (58.0%), vaccine safety (42.9%), and distrust of the vaccine (41.9%) (Table 1). Higher percentages of the reachable group than the reluctant group planned to wait to see if the vaccine is safe (54.4% vs. 35.2%) and believed that other persons need the vaccine more (28.7% vs. 12.2%). A higher percentage of the reluctant group reported a lack of trust in COVID-19 vaccines compared with the reachable group (56.2% vs. 22.3%). More respondents in the reluctant group also did not believe a vaccine is needed (36.6% vs. 10.5% of reachable group), did not think COVID-19 is much of a threat (25.9% vs. 8.6%), and did not know whether a vaccine will work (27.8% vs. 19.9%).

**Table 1:** Reasons for not getting a COVID-19 vaccine, by essential worker group and vaccination intent, United States, March–June 2021*

Reason	All groups, n = 5,308				Healthcare personnel, n = 1,308				Frontline workers, n = 2,300				Other workers, n = 1,700		
	Total	R, n = 714	RL, n = 1,059		Total	R, n = 121	RL, n = 197		Total	R, n = 316	RL, n = 437		Total	R, n = 277	RL, n = 425
Possible side effects	58.0 (55.0–60.9)	59.5 (54.7–64.2)	57.3 (53.4–61.1)		58.3 (50.6–65.7)	55.5 (44.1–66.4)	60.4 (50.2–69.9)		57.9 (53.3–62.3)	58.4 (51.0–65.5)	57.6 (51.5–63.5)		58.0 (53.3–62.6)	62.3 (54.5–69.7)	55.1 (49.2–60.9)
Wait and see if it is safe	42.9 (40.1–45.8)	**54.4 (49.5–59.3)**	35.2 (31.6–38.9)		46.3 (39.1–53.7)	**57.7 (46.3–68.5)**	41.0 (31.6–50.9)		41.4 (37.3–45.7)	**50.4 (43.5–57.3)**	35.0 (29.9–40.4)		**43.0 (38.2–47.9)**	57.6 (49.2–65.7)	32.1 (26.9–37.8)
Do not trust the vaccine	41.9 (39.0–44.8)	**22.3 (18.6–26.4)**	56.2 (52.3–59.9)		45.2 (37.7–52.8)	**21.2 (13.3–31.1)**	57.9 (48.2–67.2)		41.3 (37.0–45.6)	**24.9 (19.1–31.5)**	54.2 (48.2–60.2)		**41.0 (36.6–45.5)**	19.8 (14.4–26.3)	57.3 (51.4–63.0)
Vaccine is not needed	25.7 (23.1–28.5)	**10.5 (7.8–13.7)**	36.6 (32.8–40.5)		23.7 (17.3–31.1)	**8.5 (4.2–15.0)**	31.8 (22.6–42.1)		24.2 (20.5–28.2)	**10.8 (7.1–15.6)**	34.4 (29.0–40.2)		**28.3 (24.1–32.8)**	10.9 (6.3–17.2)	41.8 (36.0–47.7)
Concern about allergic reaction	25.0 (22.6–27.5)	26.7 (22.8–31.0)	23.9 (20.8–27.2)		23.0 (17.7–29.0)	**32.6 (22.5–44.1)**	17.3 (11.9–24.0)		26.1 (22.4–30.0)	23.5 (18.3–29.4)	28.4 (23.2–33.9)		24.9 (20.8–29.2)	28.1 (21.3–35.8)	22.6 (18.0–27.7)
Vaccine might not work	24.3 (21.8–27.0)	**19.9 (16.3–23.9)**	27.8 (24.2–31.5)		28.5 (21.6–36.2)	**17.7 (11.1–26.3)**	34.3 (24.8–44.8)		22.6 (19.3–26.3)	**17.1 (12.3–22.9)**	27.1 (22.3–32.4)		24.2 (20.2–28.6)	23.8 (17.6–30.9)	24.8 (19.6–30.5)
Others need vaccine more	19.0 (16.8–21.4)	**28.7 (24.8–32.9)**	12.2 (9.8–14.9)		14.9 (9.8–21.3)	19.7 (11.9–29.8)	12.6 (6.3–21.7)		18.2 (15.1–21.6)	**26.0 (20.1–32.5)**	12.4 (9.2–16.1)		**21.8 (18.1–26.0)**	35.1 (27.9–42.9)	11.8 (8.3–16.2)
COVID-19 is not a threat	18.7 (16.5–21.0)	**8.6 (5.9–12.0)**	25.9 (22.9–29.2)		16.3 (11.0–22.8)	**3.1 (0.8–7.9**)†	23.3 (15.5–32.6)		17.6 (14.6–21.0)	**10.4 (6.2–16.1)**	23.2 (18.9–28.0)		**21.0 (17.4–24.9)**	8.6 (4.3–15.0)	30.4 (25.3–35.9)
Do not like needles	10.4 (8.6–12.5)	12.3 (9.1–16.1)	9.2 (6.9–11.9)		8.7 (4.5–14.8)	6.6 (1.7–16.7)†	9.8 (4.3–18.4)†		11.2 (8.5–14.5)	14.7 (9.9–20.6)	8.8 (5.6–13.0)		10.4 (7.5–13.9)	11.8 (7.0–18.3)	9.3 (6.1–13.6)
Obstacles prevent vaccination	5.1 (3.8–6.8)	6.7 (4.4–9.6)	4.0 (2.4–6.3)		6.5 (2.8–12.6)†	7.5 (3.2–14.5)†	6.0 (1.4–15.9)†		4.9 (3.2–7.2)	6.2 (3.7–9.7)	3.8 (1.7–7.2)†		4.7 (2.7–7.6)	6.8 (3.0–13.1)†	3.1 (1.6–5.5)
Concerned about cost	4.6 (3.2–6.5)	**7.2 (4.5–10.8)**	2.8 (1.5–5.0)		3.8 (1.4–8.0)†	7.0 (1.6–18.2)†	2.1 (0.4–6.2)†		3.6 (2.0–6.0)	5.8 (3.0–10.1)	2.0 (0.5–5.3)		6.2 (3.4–10.2)	8.8 (3.9–16.7)†	4.2 (1.5–9.1)†
Community members are not getting vaccinated	3.1 (2.2–4.2)	2.6 (1.6–4.1)	3.4 (2.2–5.1)		1.2 (0.3–3.2)	1.3 (0.1–5.5)†	1.2 (0.2–3.9)		4.0 (2.5–6.1)	2.9 (1.3–5.4)	4.9 (2.6–8.4)		2.9 (1.7–4.6)	2.9 (1.3–5.6)	3.0 (1.3–5.6)

*Values are reported as % respondents (95% CI). n values indicate unweighted sample size/denominator. Bold text indicates statistical significance (p<0.05) between reachable and reluctant groups; reluctant group is the referent. R respondents were defined as adults who probably would or were unsure about getting a COVID-19 vaccine. RL respondents were defined as adults who probably or definitely would not get a COVID-19 vaccine. COVID-19, coronavirus disease; R, reachable; RL, reluctant.

†Estimates do not meet the National Center for Health Statistics standards of reliability (https://www.cdc.gov/nchs/data/series/sr_02/sr02_175.pdf).

Concern about COVID-19 disease was lower (38.5%) than concern about side effects from the vaccine (46.4%) among all essential workers (Appendix Table 2). Among reachable groups, 42.5% reported concern about getting COVID-19 compared with 21.8% of those in reluctant groups. Among HCP, 85.5% of reachable respondents were concerned about vaccine side effects compared with 68.8% of those in reluctant groups.

Among all essential workers, the main motivators for getting vaccinated were protection from spreading COVID-19 to family and friends (51.4%), receiving more information on effectiveness of COVID-19 vaccines (44.4%), and reducing spread of COVID-19 in the community (41.9%) (Table 2). Motivators that were higher among the reachable than the reluctant groups were increased information on vaccine safety (39.8% vs. 20.8%) and efficacy (31.1% vs. 14.1%), requirement by workplace or school (29.1% vs. 14.2%), and protection for family and friends (26.0% vs. 4.7%).

**Table 2:** Motivators for getting a COVID-19 vaccine, by essential worker group and vaccination intent, United States, March*–*June 2021*

Reason	All groups, n = 5,308				Healthcare personnel, n = 1,308				Frontline workers, n = 2,300				Other workers, n = 1,700		
	Total†	R, n = 714	RL, n = 1,059		Total	R, n = 121	RL, n = 197		Total	R, n = 316	RL, n = 437		Total	R, n = 277	RL, n = 425
Prevent COVID-19 spread to family and friends	51.4 (49.6–53.2)	**26.0 (22.0–30.3)**	4.7 (3.1–6.9)		58.0 (54.4–61.4)	**30.5 (20.3–42.3)**	7.7 (2.5–17.2)†		50.5 (47.7–53.3)	**30.7 (24.4–37.5)**	4.1 (2.2–6.8)		47.6 (44.7–50.5)	**19.1 (13.7–25.4)**	3.7 (2.0–6.3)
More information on vaccine effectiveness	44.4 (42.7–46.2)	**31.1 (26.7–35.7)**	14.1 (11.6–16.9)		46.0 (42.6–49.4)	**26.4 (17.6–36.8)**	13.7 (8.5–20.5)		44.9 (42.3–47.5)	**29.1 (23.5–35.2)**	17.1 (13.1–21.7)		42.6 (39.7–45.6)	**35.0 (27.7–42.9)**	11.0 (7.4–15.6)
Reduce COVID-19 spread in community	41.9 (40.2–43.6)	**19.4 (15.9–23.2)**	2.9 (1.7–4.7)		45.8 (42.3–49.4)	**14.5 (8.3–23.0)**	3.0 (1.1–6.5)†		42.5 (39.8–45.2)	**20.9 (16.0–26.6)**	3.2 (1.0–7.3)†		37.9 (35.0–40.9)	**19.4 (13.5–26.4)**	2.6 (1.1–5.2)
Ability to resume social activities	36.7 (35.0–38.3)	**15.5 (12.3–19.2)**	2.2 (1.3–3.3)		38.5 (35.1–42.0)	**9.0 (4.5–15.7)**	1.2 (0.2–3.8)		37.7 (35.3–40.2)	**14.3 (9.9–19.6)**	3.6 (1.9–6.1)		33.8 (31.1–36.5)	**19.3 (13.4–26.3)**	1.2 (0.4–2.8)
More severe COVID-19 cases	33.4 (31.8–35.0)	**13.7 (10.8–17.0)**	4.8 (3.4–6.6)		36.2 (32.9–39.5)	**14.4 (7.8–23.6)**	2.2 (0.7–5.4)		33.6 (31.2–36.1)	**13.1 (9.1–18.1)**	6.2 (3.5–10.0)		30.9 (28.3–33.6)	**14.0 (9.3–19.9)**	4.7 (2.7–7.5)
Ability to travel	31.3 (29.8–32.8)	**16.9 (13.8–20.5)**	6.9 (5.1–9.1)		32.5 (29.2–35.9)	15.0 (8.1–24.5)	7.4 (3.2–14.2)†		32.4 (30.1–34.7)	**16.4 (12.1–21.6)**	6.4 (4.1–9.4)		28.8 (26.2–31.4)	**18.3 (12.7–24.9)**	7.1 (4.3–10.8)
Someone I know became seriously ill or died from COVID-19	22.1 (20.8–23.5)	9.4 (6.8–12.6)	6.4 (4.6–8.5)		24.1 (21.4–27.0)	**11.7 (5.6–21.0)**†	2.7 (0.8–6.6)†		21.7 (19.7–23.9)	11.5 (6.9–17.8)	8.0 (5.2–11.6)		21.0 (18.7–23.4)	6.1 (3.6–9.6)	6.6 (3.9–10.4)
Recommend by a healthcare provider	17.2 (16.0–18.5)	**14.1 (10.9–17.9)**	3.6 (2.2–5.5)		19.9 (17.4–22.6)	**13.5 (6.3–24.2)**†	3.9 (0.8–11.0)†		16.8 (14.9–18.7)	**15.5 (10.8–21.2)**	4.2 (2.2–7.4)		15.7 (13.7–17.9)	**12.9 (7.7–19.8)**	2.8 (1.2–5.5)
Workplace or school requirement	14.9 (13.8–16.1)	**29.1 (25.0–33.5)**	14.2 (11.8–17.0)		19.9 (17.2–22.9)	**39.5 (28.6–51.2)**	10.8 (6.5–16.7)		13.7 (12.0–15.4)	**27.2 (21.6–33.5)**	17.1 (13.0–21.9)		12.8 (10.8–15.0)	**27.4 (20.9–34.7)**	13.0 (9.4–17.4)
Vaccine safety information available	14.8 (13.4–16.3)	**39.8 (35.4–44.5)**	20.8 (17.9–24.1)		11.7 (9.3–14.6)	**46.6 (35.6–57.9)**	21.2 (14.7–28.9)		14.2 (12.2–16.4)	**36.8 (30.5–43.3)**	21.7 (17.1–26.8)		18.0 (15.2–21.1)	**40.7 (33.3–48.6)**	19.7 (15.1–25.1)
Enables children back to school	14.8 (13.6–16.0)	**8.1 (5.9–10.9)**	1.5 (0.7–2.8)		16.5 (13.9–19.3)	**12.6 (5.6–23.3)**†	2.0 (0.6–5.1)		16.0 (14.2–17.8)	**8.5 (5.4–12.4)**	2.2 (0.6–5.2)		11.8 (10.0–13.8)	**6.1 (3.3–10.1)**	0.4 (0.0–1.6)
Enables me to get back to work or school	12.4 (11.2–13.7)	**4.8 (3.2–7.0)**	1.4 (0.7–2.7)		14.3 (11.7–17.3)	2.2 (0.4–6.7)†	1.7 (0.4–4.6)		14.9 (13.0–16.8)	**6.4 (3.7–10.3)**	2.0 (0.5–5.1)		7.5 (6.1–9.2)	**4.1 (1.9–7.5)**†	0.7 (0.1–2.1)
Recommended by a family member or friend	11.9 (10.8–13.0)	**5.4 (3.7–7.7)**	1.3 (0.6–2.4)		11.2 (9.0–13.7)	7.6 (2.3–17.8)†	2.2 (0.6–5.3)		13.0 (11.4–14.8)	**5.9 (3.4–9.4)**	1.7 (0.5–4.4)		10.8 (9.1–12.6)	**4.2 (2.1–7.2)**	0.3 (0.0–1.3)
See community members getting vaccinated	10.3 (9.1–11.5)	**5.2 (3.4–7.5)**	2.6 (1.3–4.6)		10.5 (8.2–13.2)	10.2 (3.7–21.3)†	2.1 (0.6–5.2)		11.7 (9.8–13.8)	5.3 (3.0–8.6)	4.5 (1.7–9.4)†		8.2 (6.7–9.9)	**3.2 (1.4–6.0)**	0.7 (0.1–2.2)
Large increase in COVID-19 cases in my area	3.1 (2.5–3.9)	**10.1 (7.7–13.0)**	3.2 (2.0–4.9)		2.3 (1.2–4.1)	**13.5 (6.4–24.1)**†	2.1 (0.5–5.3)		3.2 (2.2–4.4)	7.8 (5.1–11.4)	5.1 (2.6–8.8)		3.7 (2.5–5.4)	**11.4 (7.2–17.1)**	1.8 (0.7–3.7)
None of the above	19.9 (18.3–21.5)	**14.1 (11.1–17.6)**	55.9 (52.1–59.6)		15.6 (12.3–19.4)	**10.7 (5.8–17.7)**	54.1 (44.3–63.6)		17.7 (15.4–20.1)	**11.9 (8.1–16.7)**	52.7 (46.6–58.7)		26.2 (23.3–29.2)	**17.8 (12.1–24.9)**	60.5 (54.5–66.3)

*Values are reported as % respondents (95% CI). n values indicate unweighted sample size/denominator. Bold text indicates statistical significance (p<0.05) between reachable and reluctant groups; reluctant group is the referent. R respondents were defined as adults who probably would or were unsure about getting a COVID-19 vaccine. RL respondents were defined as adults who probably or definitely would not get a COVID-19 vaccine. COVID-19, coronavirus disease; R, reachable; RL, reluctant.

†Estimates do not meet the National Center for Health Statistics standards of reliability (https://www.cdc.gov/nchs/data/series/sr_02/sr02_175.pdf).

## Conclusions

Despite their increased risk for COVID-19 exposure, only about 70% of essential workers included in the sample received >1 vaccine dose by early June 2021, similar to the 69% of all adults in the sample population during the same time period (data not shown). Over the 6 waves of data collection, HCP had the highest vaccination coverage (66.6%); those in the other essential worker group had the lowest vaccination coverage (45%) during March–June 2021, and one quarter were reluctant to get COVID-19 vaccinations. Consistent with another study (*11*), we found that younger adults and those who have lower education or income levels are more vaccine hesitant. 

The first limitation of our study is that although the panel recruitment survey methodology and data weighting were designed to produce nationally representative results, respondents might not be fully representative of the general US adult population. Vaccination coverage among respondents was self-reported and could be subject to recall or social desirability bias. Data were combined across multiple survey waves, which might overaverage any recent changes in vaccination coverage and intent. Finally, state-specific vaccine prioritization varied during the data collection period, which might have affected vaccination coverage responses to items related to attitudes, behaviors, and perceptions.

Among essential workers in this sample, predominant motivators for getting vaccinated were protecting family and friends, gaining more information about the safety and effectiveness of vaccinates, and preventing community spread. These data suggest that clear, consistent messages from healthcare providers, public health officials, and immunization partners about the safety and effectiveness of the vaccine could increase vaccination coverage and vaccine confidence more broadly (*12*). In addition, framing messages in terms of benefits such as protecting family and friends; being able to travel; and resuming work, school, and social activities might further boost immunization coverage and confidence (*12*). 

Among unvaccinated essential workers, nearly 60% were worried about vaccine side effects. Connecting employers and employees to credible resources on vaccine safety and expected side effects might improve vaccination coverage among essential workers. Implementing interventions to mitigate barriers to vaccination, such as flexible scheduling, paid time off for vaccination and illness resulting from side effects, on-site vaccination, and walk-in clinics, also could improve vaccination coverage. 

In conclusion, our findings suggest public health officials and other leaders should differentiate between continued challenges in accessing vaccines for all populations from behavioral factors associated with vaccination. To reach vaccination goals for essential workers and everyone in the community, healthcare providers, public health officials, and immunization partners should consider KABs when tailoring messages and strategies to increase vaccination uptake and confidence, especially at local community levels.

Appendix Additional information on coronavirus disease vaccination coverage, intent, and knowledge, attitudes, and behaviors among healthcare workers, United States.
