# Supplementary material for: COVID-19 Vaccination Coverage, Intent, Knowledge, Attitudes, and Beliefs among Essential Workers, United States
Source: Emerg Infect Dis. 2021 Nov;27(11):2908–13. doi: 10.3201/eid2711.211557 (PMC8544962; doi:10.3201/eid2711.211557)
Supplement: Appendix — Additional information on coronavirus disease vaccination coverage, intent, and knowledge, attitudes, and behaviors among healthcare workers, United States. [file 21-1557-Techapp-s1.pdf]

Article DOI: https://doi.org/10.3201/eid2711.211557

COVID-19 Vaccination Coverage, Intent, Knowledge, Attitudes, and Beliefs among Essential Workers, United States

Appendix

Additional Methods

In December 2020, the Advisory Committee on Immunization Practices (ACIP) issued recommendations prioritizing persons for vaccination in a series of phases for COVID-19 vaccination (*1*). ACIP recommendations prioritized healthcare personnel (HCP) and long-term care facility residents in Phase 1a, persons aged ≥75 years and non-healthcare frontline essential workers in Phase 1b, and persons aged 65–74 years, persons with high-risk medical conditions aged 16–64 years, and essential workers who were not in Phase 1a or 1b into Phase 1c (*1*).

We analyzed data from 2 nationally representative household panel omnibus surveys collected during March 2, –June 5, 2021, Ipsos KnowledgePanel (*2*) and NORC AmeriSpeak (*3*). For Ipsos, data were fielded during March 5–7, March 19–29, April 9–11, April 23–May 2, May 7–9, and May 21–31, 2021. For NORC, data were collected during March 11–15, March 25–29, April 15–19, April 29–May 4, May 13–17, and May 27–June 2, 2021 (Figure 1).

We examined the following sociodemographic characteristics: age groups were 18–34 years, 35–49 years, 50–64 years, or ≥65 years; race/ethnicity were reported as non-Hispanic (NH) White, NH Black, Hispanic, NH Asian persons, or non-Hispanic other/multiple races persons; annual household income was reported as <$35,000, $35,000–$49,999, $50,000–$74,999, or ≥$75,000; health insurance status was reported as yes or no; marital status was reported as married or living with a partner, separated or divorced or widowed, or never married; urban versus rural status was reported as urban, suburban, or rural; and yes or no for any underlying conditions. Underlying condition status was ascertained through the following question: Have you ever been told by a doctor or other health professional that you had any of

the following medical conditions? Respondents could select any of the listed conditions, which were derived from a list of conditions that increase risk for severe illness from COVID-19 (*4*) (Appendix Table 2).

We weighted data to ensure representation of the US population by using demographic benchmarks from the 2019 American Community Survey (*5*), such as age, sex, race/ethnicity, education, and income. We also used the 2020 Current Population Survey (*6*) to determine respondent residence in a Metropolitan Statistical Area (MSA) or non-MSA, that is rural area.

This activity complied with Centers for Disease Control and Prevention (CDC) policy on human subjects research (*7*). The study also complies with federal laws, including 45 CFR part 46.102(l) (2) (*8*); 21 CFR part 56 (*9*); 42 USC Section 241(d) (*10*); 5 USC Section 552a (*11*); and 44 USC Section 3501 et seq (*12*).

Essential Worker Group Categorization

We categorized respondents according to ACIP's essential worker groups (*13*). We determined essential workers by using the following question: Which best describes the current primary location/setting where you work or volunteer? Respondents could select 1 response.

We categorized respondents as HCP if they replied that their work or volunteer duties fell into any of the following 3 categories: healthcare, such as hospital, doctor, dentist, or mental health specialist office, outpatient facility, long-term care, home health care, pharmacy, or medical laboratory; social service, such as child, youth, family, elderly, or disability services; or death care, such as funeral home, crematory, or cemetery.

We categorized respondents as nonhealthcare frontline essential workers if they replied that their work or volunteer duties fell into any of the following 10 categories: education, at a prekindergarten or kindergarten–12 school setting or in childcare; other education, such as business or technical school, college, or university; first response, such as police or fire protection, or emergency relief services; correctional facility, such as jail, prison, detention center, or reformatory; food and beverage stores, such as grocery store, warehouse club, supercenter, convenience store, specialty food store, bakery, or liquor store; agriculture, forestry, fishing, or hunting; food manufacturing facility, such as meat processing, produce packing, or food or beverage manufacturing; non-food manufacturing facility, such as metals, equipment and

machinery, or electronics; public transit, such as bus, commuter rail, subway, or school bus; or United States Postal Service. We categorized respondents as nonessential when they reported any work setting other than those classified as essential.

Vaccination Status, Intent, and Knowledge, Attitudes, and Beliefs Questions Toward Vaccination

We assessed vaccination status, intent, and knowledge, attitudes, and beliefs (KABs) through survey questions. We assessed vaccination status by using the following question: Have you received at least one dose of a COVID-19 vaccine? Respondents could select yes or no.

Among adults who were not vaccinated, we asked: How likely are you to get a COVID-19 vaccine? Response options were definitely will get a vaccine, probably will get a vaccine, unsure about getting a vaccine, probably will not get a vaccine, and definitely will not get a vaccine. We categorized respondents as reachable if they probably would or were unsure about getting a vaccine. We categorized respondents as reluctant if they probably or definitely would not get a vaccine.

We assessed the following KABs about the COVID-19 vaccine: reasons for not getting vaccinated, barriers in getting a vaccine, motivators for getting a vaccine, concerns about getting COVID-19, and concerns about having any side effects from a COVID-19 vaccine.

We asked about reasons for not getting vaccinated for all respondents except those who already were vaccinated or definitely planned to get vaccinated. We used the question, Which of the following, if any, are reasons that you (only probably will get/are unsure about getting/probably won't get/definitely won't get/won't receive all required doses of) a/the COVID-19 vaccine?

We assessed barriers in getting a vaccine by asking everyone who did not get vaccinated, What makes it difficult for you to get a COVID-19 vaccine? Possible responses were nothing; side effects will interfere with daily activities; difficult to make online appointment; not sure; do not have time off work; cannot find convenient appointment time; other (Appendix Table 2).

We asked all respondents, regardless of vaccination status, about motivators for getting vaccinated. We used the following question to assess vaccination barriers: Which of the

following [made/would make] you [definitely plan/more likely] to get a COVID-19 vaccine? Possible responses included prevent COVID-19 spread to family and friends; get more information on vaccine effectiveness; reduce COVID-19 spread in community; ability to resume social activities; more severe COVID-19 cases in the community; and ability to travel (Table 2).

We asked respondents whether they were concerned about getting COVID-19; How concerned are you about getting COVID-19? We also asked whether they were concerned about vaccine side effects; How concerned are you about having any side effects from the COVID-19 vaccine? Possible responses to COVID-19 and vaccination concerns included very concerned, somewhat concerned, slightly concerned, and not concerned. We categorized respondents as having concerns when they reported being very concerned or somewhat concerned. We did not include results for slightly concerned or not concerned in this report.

References

1. Centers for Disease Control and Prevention. COVID-19 ACIP vaccine recommendations [cited 2021 Apr 21]. https://www.cdc.gov/vaccines/hcp/acip-recs/vacc-specific/covid-19.html

2. Ipsos. KnowledgePanel: a methodological overview [cited 2021 Mar 30]. https://www.ipsos.com/sites/default/files/ipsosknowledgepanelmethodology.pdf

3. NORC. Amerispeak omnibus [cited 2021 Mar 30]. https://amerispeak.norc.org/our-capabilities/Pages/AmeriSpeak-Omnibus.aspx

4. Centers for Disease Control and Prevention. COVID-19: people with certain medical conditions [cited 2021 May 25]. https://www.cdc.gov/coronavirus/2019-ncov/need-extra-precautions/people-with-medical-conditions.html

5. United States Census Bureau. American community survey: data profiles 2019 [cited 2021 May 1]. https://www.census.gov/acs/www/data/data-tables-and-tools/data-profiles

6. United States Census Bureau. 2020 State-based metropolitan and micropolitan statistical area maps [cited 2021 May 1]. https://www.census.gov/geographies/reference-maps/2020/demo/state-maps.html

7. Centers for Disease Control and Prevention. Human participant protection in CDC research [cited 2021 May 12]. https://www.cdc.gov/os/integrity/hrpo/index.htm

8. Code of Federal Regulations. 45 CFR part 46: protection of human subjects [cited 2021 Mar 15]. https://ecfr.federalregister.gov/current/title-45

9. Code of Federal Regulations. 21 CFR part 56: institutional review boards [cited 2021 Mar 15]. https://ecfr.federalregister.gov/current/title-21/chapter-I/subchapter-A/part-56

10. United States Code. 42 USC section 241(d): research and investigations [cited 2021 Mar 15]. https://www.govinfo.gov/app/details/USCODE-2011-title42/USCODE-2011-title42-chap6A-subchapII-partA-sec241/summary

11. United States Code. 5 USC section 552a: records maintained on individuals [cited 2021 Mar 15]. https://www.govinfo.gov/app/collection/uscode/2019/title5/partI/chapter5/subchapterII/Sec.%20552a

12. United States Code. 44 USC section 3501: federal information policy [cited 2021 Mar 15]. https://www.govinfo.gov/app/collection/uscode/2019/title44/chapter35/subchapterI

13. Centers for Disease Control and Prevention. Interim list of categories of essential workers mapped to standardized industry codes and titles [cited 2021 May 12]. https://www.cdc.gov/vaccines/covid-19/categories-essential-workers.html

Appendix Table 1. Proportion of reachable and reluctant adults, by essential worker status and socioeconomic characteristics, United States, March–June 2021*

Characteristics	All groups†		Healthcare personnel		Frontline workers		Other workers	
	**R, n = 714**	**RL, n = 1,059**	**R, n = 121**	**RL, n = 197**	**R, n = 316**	**RL, n = 437**	**R, n = 277**	**RL, n = 425**
All adults ≥18 y	**15.3 (14.1–16.6)**	21.5 (20.1–22.9)	**9.3 (7.5–11.4)**	18.3 (15.4–21.5)	**15.6 (13.7–17.6)**	20.4 (18.2–22.7)	**19.6 (17.0–22.4)**	25.5 (23.0–28.1)
Age groups, y								
18–34	**18.8 (16.3–21.6)**	25.0 (22.2–28.0)	**11.2 (7.8–15.5)**	25.4 (19.2–32.4)	18.8 (15.2–22.8)	21.4 (17.5–25.7)	26.2 (20.2–32.8)	30.8 (25.8–36.1)
35–49	**15.8 (13.7–18.1)**	24.1 (21.7–26.7)	**10.3 (6.9–14.7)**	19.0 (14.3–24.5)	**16.2 (12.7–20.3)**	24.1 (20.4–28.1)	**19.2 (15.2–23.6)**	27.7 (23.3–32.5)
50–64	**12.9 (10.9–15.1)**	17.4 (15.1–19.8)	7.7 (4.7–11.6)	11.9 (8.2–16.6)	13.5 (10.4–17.0)	17.1 (13.4–21.3)	**15.2 (11.7–19.4)**	20.9 (17.3–24.9)
≥65	5.8 (3.6–8.8)	9.6 (6.6–13.3)	3.7 (1.4–8.0)‡	5.9 (2.1–12.8)‡	4.1 (1.2–9.8)‡	12.5 (7.5–19.1)	11.4 (5.6–19.8)	10.2 (5.1–17.7)
Sex								
M	**16.8 (14.8–18.9)**	20.9 (19.0–23.0)	**6.1 (3.9–9.1)**	13.8 (9.9–18.6)	17.2 (14.4–20.2)	20.5 (17.7–23.6)	20.6 (17.0–24.5)	24.3 (21.1–27.7)
F	**13.8 (12.1–15.6)**	22.0 (19.9–24.2)	**10.8 (8.4–13.6)**	20.4 (16.6–24.6)	**14.0 (11.4–16.8)**	20.3 (17.1–23.8)	**17.9 (14.1–22.1)**	27.5 (23.3–32.0)
Race/ethnicity								
Non-Hispanic White	**14.1 (12.6–15.6)**	22.9 (21.2–24.7)	**8.3 (6.1–10.8)**	17.3 (13.8–21.3)	**14.7 (12.5–17.2)**	22.4 (19.7–25.2)	**17.0 (14.1–20.3)**	27.2 (24.3–30.2)
Non-Hispanic Black	19.1 (14.8–23.9)	20.7 (16.6–25.2)	15.5 (9.7–22.9)	17.0 (10.3–25.7)	22.0 (15.4–29.9)	19.3 (13.5–26.3)	18.2 (10.2–28.8)	28.6 (19.4–39.2)
Hispanic	16.8 (13.6–20.3)	19.4 (15.6–23.7)	**8.8 (4.9–14.4)**	24.8 (16.4–34.9)	14.4 (10.1–19.7)	16.2 (11.2–22.4)	26.9 (19.7–35.2)	19.2 (13.2–26.4)
Non-Hispanic Other/multiple races	15.4 (10.7–21.2)	17.0 (12.4–22.4)	5.8 (1.8–13.6)‡	12.7 (5.2–24.5)‡	14.0 (8.0–22.0)	16.9 (10.9–24.5)	26.7 (15.3–40.8)	21.1 (11.0–34.7)
Educational Status								
High school or less	**21.9 (19.0–25.2)**	28.1 (25.0–31.4)	**12.2 (7.7–18.0)**	29.5 (21.7–38.4)	23.5 (19.0–28.5)	26.4 (21.7–31.5)	25.8 (20.2–32.1)	29.6 (24.9–34.7)
Some college or college graduate	**14.1 (12.7–15.6)**	20.9 (19.2–22.6)	**9.1 (7.0–11.5)**	16.8 (13.7–20.3)	**14.2 (12.1–16.5)**	19.9 (17.4–22.7)	**17.7 (15.0–20.7)**	25.0 (21.9–28.2)
Above college graduate	**5.0 (3.5–6.9)**	9.4 (7.3–11.9)	6.0 (3.2–10.1)	6.7 (3.6–11.3)	**3.6 (2.0–5.9)**	9.9 (7.0–13.4)	7.0 (3.7–11.9)	12.8 (7.3–20.4)
Annual household income, $US								
<25,000	21.8 (17.2–26.9)	26.3 (21.0–32.1)	**9.9 (5.4–16.2)**	26.9 (16.5–39.5)	24.4 (17.3–32.8)	25.4 (18.3–33.6)	37.0 (25.5–49.7)	27.4 (18.4–37.9)
25,000–49,999	**18.4 (15.1–22.2)**	25.8 (22.4–29.5)	12.3 (7.5–18.7)	17.6 (12.4–23.8)	**17.7 (13.2–23.1)**	28.6 (23.2–34.4)	24.2 (17.4–32.0)	28.3 (22.2–35.0)
50,000–74,999	**15.4 (12.6–18.5)**	24.4 (21.1–28.0)	**9.1 (5.0–15.0)**	24.8 (17.7–32.9)	17.4 (12.9–22.8)	21.8 (17.2–27.0)	**16.9 (12.2–22.4)**	27.5 (21.4–34.2)
≥75,000	**12.2 (10.7–13.9)**	17.3 (15.6–19.1)	**7.9 (5.7–10.7)**	13.1 (10.0–16.8)	**11.4 (9.3–13.7)**	14.8 (12.4–17.5)	**16.4 (13.1–20.0)**	23.3 (20.2–26.7)
Insurance status								
Insured	**14.3 (13.0–15.6)**	20.2 (18.7–21.7)	**8.7 (6.9–10.8)**	17.2 (14.3–20.4)	**13.7 (11.9–15.7)**	19.2 (17.0–21.6)	**19.3 (16.6–22.2)**	23.8 (21.2–26.5)
Not insured	**23.5 (18.2–29.5)**	32.3 (26.1–38.9)	11.0 (4.5–21.5)‡	33.6 (19.7–49.9)‡	28.2 (19.8–37.8)	28.2 (19.8–38.0)	25.6 (15.9–37.4)	37.7 (26.8–49.6)
Marital status								
Married/Living with partner	**13.3 (11.9–14.7)**	21.7 (20.0–23.6)	**8.3 (6.2–10.9)**	17.2 (13.8–21.0)	**12.8 (10.8–15.1)**	22.1 (19.1–25.2)	**17.5 (14.6–20.7)**	24.5 (21.4–27.9)
Separated/divorced/widowed	**12.4 (9.1–16.3)**	23.2 (19.7–27.0)	9.3 (5.2–14.9)	15.3 (9.1–23.6)	**12.9 (7.4–20.3)**	22.4 (17.1–28.4)	**14.4 (8.7–21.8)**	30.9 (24.2–38.3)
Never married	20.3 (17.5–23.4)	20.2 (17.4–23.2)	**11.1 (7.3–15.9)**	21.6 (15.4–28.9)	21.6 (17.4–26.3)	16.7 (13.0–20.9)	26.7 (21.0–33.0)	24.6 (19.7–29.9)
MSA								
Urban	15.7 (13.4–18.2)	16.5 (14.4–18.7)	8.9 (5.9–12.8)	13.9 (9.8–18.9)	14.6 (11.2–18.5)	15.2 (12.2–18.7)	23.7 (18.4–29.6)	20.8 (16.9–25.2)
Suburban	**14.4 (12.7–16.2)**	21.7 (19.6–23.9)	**8.8 (6.5–11.7)**	19.4 (15.3–24.1)	15.8 (12.9–19.0)	19.7 (16.4–23.2)	**16.7 (13.5–20.4)**	25.9 (22.4–29.7)
Rural	**16.9 (14.3–19.9)**	29.3 (26.1–32.8)	**11.6 (6.8–18.0)**	24.1 (16.1–33.7)	**16.7 (13.3–20.7)**	30.3 (25.6–35.3)	**21.0 (15.6–27.3)**	31.5 (25.8–37.7)
Underlying conditions								
Y	**15.3 (13.6–17.2)**	21.4 (19.5–23.5)	**9.0 (6.6–11.9)**	20.4 (16.3–25.0)	**15.8 (13.0–18.8)**	20.8 (17.8–24.0)	20.1 (16.6–23.9)	23.1 (19.8–26.7)
N	**15.2 (13.3–17.2)**	21.7 (19.6–24.0)	**9.9 (7.1–13.3)**	15.9 (12.0–20.6)	**15.4 (12.7–18.5)**	20.3 (17.3–23.7)	**18.7 (15.0–22.8)**	28.2 (24.4–32.2)

*Values are reported as % (95% CI). Bold text indicates statistical significance (p<0.05) between reachable and reluctant groups; reluctant group is the referent. Reachable (R) respondents were defined as adults who probably would or were unsure about getting a COVID-19 vaccine. Reluctant (RL) respondents were defined as adults who probably or definitely would not get a COVID-19 vaccine. COVID-19, coronavirus disease; MSA, metropolitan statistical area; R, reachable; RL, reluctant.
†n = unweighted sample size/denominator.
‡Estimates do not meet the National Center for Health Statistics standards of reliability (https://www.cdc.gov/nchs/data/series/sr_02/sr02_175.pdf).

Appendix Table 2. COVID-19 vaccination attitudes and barriers to vaccination by essential worker group and vaccination intent, United States, March–June 2021*

	All groups, n = 5,308			Healthcare personnel, n = 1,308			Frontline workers, n = 2,300			Other workers, n = 1,700		
Barriers	Total†	R, n = 714	RL, n = 1,059	Total	R, n = 121	RL, n = 197	Total	R, n = 316	RL, n = 437	Total	R, n = 277	RL, n = 425
Concerned about getting COVID-19‡	38.5 (36.8–40.2)	**42.5 (37.8–47.3)**	21.8 (18.5–25.3)	38.9 (35.5–42.3)	**48.8 (37.3–60.4)**	25.0 (16.5–35.0)	40.0 (37.3–42.6)	**43.6 (36.8–50.6)**	23.5 (18.7–28.7)	36.2 (33.3–39.1)	**38.9 (31.1–47.1)**	18.2 (13.5–23.6)
Concerned about vaccine side effects‡	46.4 (44.7–48.1)	73.1 (68.5–77.4)	70.9 (67.2–74.5)	41.0 (37.6–44.5)	**85.5 (78.0–91.3)**	68.8 (59.1–77.5)	47.4 (44.9–50.0)	72.7 (65.9–78.8)	73.7 (67.8–79.0)	49.3 (46.4–52.2)	69.0 (60.7–76.4)	69.1 (63.5–74.3)
What makes it difficult for you to get a COVID-19 vaccine?§												
Nothing, it is not difficult	52.0 (49.3–54.6)	**40.7 (36.1–45.5)**	71.3 (67.5–74.9)	55.2 (48.4–61.7)	**44.5 (33.5–55.9)**	69.7 (59.0–79.1)	50.8 (46.6–54.9)	**40.2 (33.4–47.3)**	68.6 (62.8–74.0)	51.8 (47.7–55.9)	**39.9 (32.4–47.8)**	75.2 (69.6–80.2)
Side effects will interfere with daily activities	16.5 (13.8–19.4)	**25.2 (19.6–31.4)**	11.7 (8.5–15.6)	13.4 (7.5–21.4)	21.8 (11.7–35.1)	9.7 (2.4–24.3)¶	18.3 (14.1–23.3)	**26.9 (18.1–37.3)**	12.4 (8.4–17.4)	16.0 (12.1–20.6)	**24.6 (16.0–35.1)**	12.1 (7.4–18.5)
Difficult to make online appointment	7.4 (6.1–8.9)	**9.1 (6.7–12.1)**	2.4 (1.3–4.1)	7.6 (4.9–11.1)	9.2 (4.1–17.3)¶	3.7 (1.3–7.8)¶	7.0 (5.3–9.2)	**7.9 (5.1–11.6)**	2.0 (0.9–3.8)	7.7 (5.4–10.6)	**10.5 (6.0–16.6)**	2.2 (0.3–7.0)¶
Not sure	5.8 (4.3–7.7)	7.4 (4.4–11.5)	6.2 (4.0–9.0)	7.3 (3.4–13.2)¶	11.8 (3.2–27.8)¶	6.4 (2.0–14.7)¶	5.7 (3.5–8.8)	9.0 (4.5–15.7)	5.7 (2.6–10.7)¶	5.3 (3.2–8.3)	3.7 (0.9–9.8)¶	6.5 (3.4–11.2)
Do not have time off work	5.4 (4.1–6.9)	6.0 (3.7–9.1)	3.2 (1.7–5.3)	3.3 (1.8–5.7)	2.4 (0.5–7.0)¶	1.3 (0.2–4.0)	5.0 (3.4–7.1)	6.1 (3.4–9.9)	3.8 (1.6–7.4)¶	6.7 (4.2–10.1)	7.2 (2.8–14.6)¶	3.6 (1.0–8.6)¶
Cannot find convenient appointment time	5.3 (3.6–7.4)	**9.0 (5.3–14.0)**	1.5 (0.3–4.0)	6.8 (3.0–12.9)¶	**16.8 (6.0–34.1)¶**	0.7 (0.0–4.2)	3.4 (2.0–5.4)	3.8 (1.4–8.0)¶	1.5 (0.4–3.9)	6.4 (3.4–10.8)	**11.7 (5.3–21.6)¶**	1.9 (0.0–9.9)¶
Other#	29.8 (26.7–33.1)	**36.2 (30.5–42.2)**	19.4 (15.5–23.8)	34.0 (26.1–42.7)	37.1 (24.4–51.3)	25.7 (15.0–39.2)	28.6 (24.1–33.5)	**32.3 (24.0–41.5)**	20.3 (14.9–26.7)	28.8 (24.1–34.0)	**40.1 (30.3–50.5)**	14.9 (10.1–20.7)

*Values are reported as % (95% CI). Bold text indicates statistical significance (p<0.05) between reachable and reluctant groups; reluctant group is the referent. Reachable (R) respondents were defined as adults who probably would or were unsure about getting a COVID-19 vaccine. Reluctant (RL) respondents were defined as adults who probably or definitely would not get a COVID-19 vaccine. COVID-19, coronavirus disease; R, reachable; RL, reluctant.
†n = unweighted sample size/denominator.
‡Asked of all respondents.
§Ask only of respondents who have not received at least one COVID-19 vaccine.
¶Estimates do not meet the National Center for Health Statistics standards of reliability (https://www.cdc.gov/nchs/data/series/sr_02/sr02_175.pdf).
#Other barriers included being too busy to get vaccinated, not knowing where to go to get vaccinated, being ineligible to get vaccinated due to a medical reason, inconvenient hours of operation, long waiting times, distance, transportation, childcare issues, physical limitation, or other reasons.
